# Enhancing Form Stability: Shrink‐Resistant Hydrogels Made of Interpenetrating Networks of Recombinant Spider Silk and Collagen‐I

**DOI:** 10.1002/adhm.202500311

**Published:** 2025-03-27

**Authors:** Xuen J. Ng, Tilman U. Esser, Vanessa T. Trossmann, Christoph Rudisch, Maren Fiedler, Kaveh Roshanbinfar, Zan Lamberger, Philipp Stahlhut, Gregor Lang, Thomas Scheibel, Felix B. Engel

**Affiliations:** ^1^ Chair of Biomaterials University of Bayreuth Prof.‐Rüdiger‐Bormann‐Str. 1 95447 Bayreuth Germany; ^2^ Experimental Renal and Cardiovascular Research Department of Nephropathology Institute of Pathology and Department of Cardiology Friedrich‐Alexander‐Universität Erlangen‐Nürnberg (FAU) Kussmaulallee 12 91054 Erlangen Germany; ^3^ Department for Functional Materials in Medicine and Dentistry University Hospital of Würzburg Pleicherwall 2 D‐97070 Würzburg Germany; ^4^ Bayreuth Center for Colloids and Interfaces (BZKG) University of Bayreuth 95447 Bayreuth Germany; ^5^ Bavarian Polymer Institute (BPI) University of Bayreuth 95447 Bayreuth Germany; ^6^ Bayreuth Center for Molecular Biosciences (BZMB) University of Bayreuth 95447 Bayreuth Germany; ^7^ Bayreuth Center for Material Science (BayMAT) University of Bayreuth 95447 Bayreuth Germany; ^8^ Faculty of Medicine University of Würzburg 97080 Würzburg Germany

**Keywords:** biopolymer, cardiac tissue engineering, compaction, heart, shrinkage

## Abstract

Tissue engineering enables the production of tissues and organ‐like structures as models for drug testing and mechanistical studies or functional replacements for injured tissues. Available cytocompatible materials are limited in number, suffer from insufficient mechanical properties, and cells interacting with them often cause construct shrinkage. As shape is important for function, identifying cytocompatible, shrink‐resistant materials are a major aim. Here, it is shown that hydrogels made of interpenetrating networks of collagen‐I and recombinant spider silk protein eADF4(C16)‐RGD nanofibrils exhibit synergistic and tunable mechanical properties. Composite hydrogels allow cell adhesion and spreading and are resistant to shrinkage mediated by fibroblasts, C2C12 myoblasts, and human induced pluripotent stem cell (hiPSC)‐derived cardiomyocytes. Myoblasts differentiate and fuse into myotubes, and hiPSC‐cardiomyocytes can be cultured long‐term, show spontaneous contractions, and remain drug responsive. Collectively, a novel composite material is developed to overcome the challenge of post‐fabrication matrix shrinkage conferring high shape fidelity suitable for tissue engineering.

## Graphical abstract

A shrinkage‐resistant hydrogel is developed to confer post‐fabrication shape fidelity. The hydrogel, based on recombinant spider silk protein eADF4(C16)‐RGD and collagen‐I, exhibits tunable mechanical properties and shrink‐resistance in the presence of fibroblasts as well as muscle cells. The hydrogel is suitable for muscle engineering allowing cell differentiation, muscle function, and drug testing.

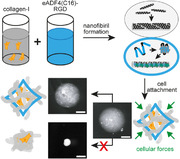

## 1 Introduction

Tissue engineering is a promising approach for generating in vitro models for drug testing and mechanistical studies or functional replacements for injured tissues. In order to generate tissues that are as similar to native tissue as possible, materials allowing cell adhesion as well as proliferation and/or differentiation and providing suitable mechanical properties have to be combined with the cell types present in the corresponding native tissue. As shape is very important for function,^[^ ^1^ ^]^ a major aim is to engineer materials that allow to fabricate hierarchically structured tissue constructs, and at the same time, prevent unwanted post‐fabrication changes in construct shape. This is a significant challenge, as most of the currently used materials are remodeled by the encapsulated cells, often resulting in cell‐induced construct shrinkage and thus, a very poor shape fidelity.

**Figure 1:**
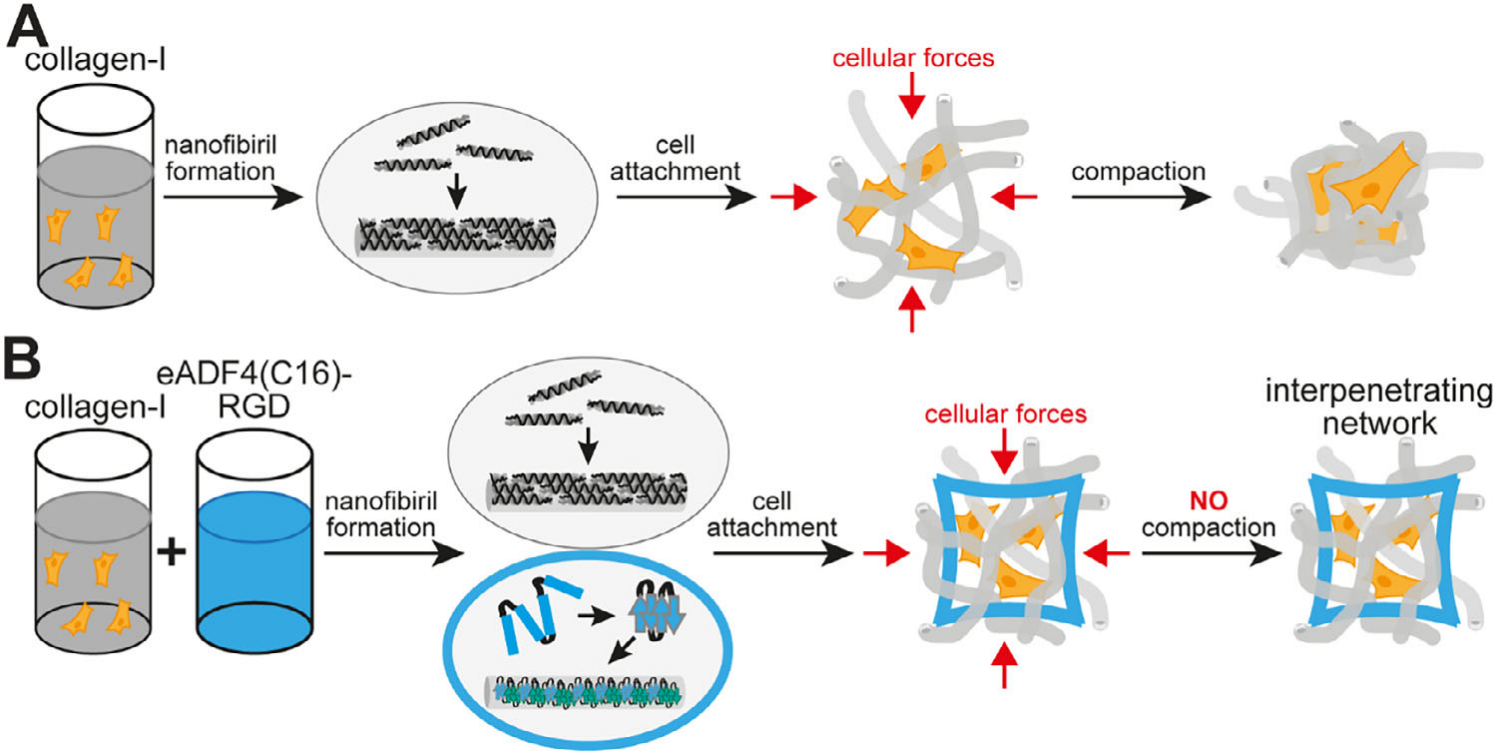
Interpenetrating networks prevent cell‐mediated shrinkage of collagen‐I‐based hydrogels. A) Collagen‐I solution self‐assembles into supramolecular nanofibrils after adjusting pH and salt concentration, forming hydrogels. Collagen‐I hydrogels are compacted by cells post‐adhesion due to traction forces through focal adhesions connected to their actin cytoskeletons. B) Hypothesis: IPNs based on recombinant spider silk eADF4(C16)‐RGD and collagen‐I prevent cell‐mediated shrinkage, whereby eADF4(C16)‐RGD provides rigidity and stability as primary network, and interlocked rat tail collagen‐I constitutes as ductile secondary network and cell interaction site.

Hydrogels, strongly hydrated polymer networks, are of particular interest as scaffolds for tissue generation, as they provide a hierarchical structure from the nano‐ to micro‐regime, and maintain high amounts of water, mimicking the cellular environment of native tissue. As the extracellular matrix (ECM) of the human body consists mainly of collagen (≈25% of our body protein mass),^[^ ^2^ ^]^ collagen‐I is widely used for tissue engineering and 3D cell culture.^[^ ^3^ ^]^ Collagen‐I is highly cytocompatible, whereby cells bind via α_1_ß_1_ and α_2_ß_1_ integrins to GFOGER domains in collagen‐I^[^ ^4^ ^]^ and exert traction forces through focal adhesions connected to their actin cytoskeletons, resulting in mechanotransduction.^[^ ^5^ ^]^ However, hydrogels made of ECM protein‐derived biopolymers, especially collagen‐I, suffer from shrinkage due to these traction forces in the presence of fibroblasts but also other cell types, also known as compaction, which is the drastic reduction in volume and size of hydrogel constructs (**Figure** **1**A).^[^ ^6^ ^]^ Therefore, hydrogels have to be stabilized using, for example, chemical cross‐linking to maintain shape fidelity. Lotz et al. addressed this issue in the context of full‐thickness skin equivalents. They chemically cross‐linked their collagen‐I‐based dermal layer with a non‐cytotoxic four‐arm succinimidyl glutarate polyethylene glycol (PEG‐SG), which resulted in maintenance of the initial construct surface area in the presence of dermal fibroblasts (2 × 10^5^ per mL ink).^[^ ^7^ ^]^ Yet, crosslinking prevented collagen fibril formation and decreased degradability. Brunel et al. addressed the issue of construct shrinkage in the context of repair and regeneration of corneal defects. They modified bovine collagen‐I with azide groups and crosslinked it with 4‐arm polyethylene glycol molecules containing bicyclononyne end groups (SPAAC Collagen).^[^ ^8^ ^]^ This allowed spreading of corneal mesenchymal stem cells (3 × 10^6^ per mL ink), while maintaining stability against cell‐induced contraction of the overall fabricated construct. However, SPAAC collagen had to be crosslinked for 2 h at room temperature, which limits its application. Finally, chemical cross‐linking usually recruits amino acid functional groups, also required for cell‐interactions, leading to adverse effects on the materials’ bioactivity.^[^ ^9^ ^]^

Another proposed approach to improve the shape fidelity of collagen‐based hydrogels is to combine collagen‐I with nano‐hydroxyapatite and mesoporous bioactive glass nanoparticles.^[^ ^10^ ^]^ However, their application is limited to hard tissues, and the effect of cells on shape fidelity was not tested. In addition, it has recently been proposed to reinforce 3D‐bioprinted constructs post‐fabrication by oxidized sucrose‐mediated crosslinking in the case of 3D‐bioprinted cardiac constructs.^[^ ^11^ ^]^ While this approach clearly improves the tissue's robustness, it remains unclear if this approach would prevent remodeling and/or shrinkage of the constructs by fibroblasts, which are essential for optimal tissue function^[^ ^12^ ^]^ but are also the most active cells in regards of ECM remodeling.^[^ ^12^, ^13^ ^]^

Therefore, the use of composite hydrogels comprising (bio)polymers with complementary properties is promising to tune hydrogel toughness. One further solution to prevent construct shrinkage in this context is the enhancement of hydrogels through interpenetrating networks (IPNs). Dhand et al. defined IPNs as follows “IPN hydrogels are formed by the combination of two or more topologically interlocking crosslinked polymer chains. The resulting two or more polymer networks within these IPN hydrogels are mutually independent, yet held together by internetwork entanglement. This approach increases the functionality of hydrogels when compared with single network (SN) hydrogel designs”.^[^ ^14^ ^]^ The cross‐linking of the IPN can be induced or occurs through self‐assembly, whereby the cross‐linking of the individual components can either happen simultaneously or sequentially, with one hydrogel network being formed first and the second one being infused into the primary network.^[^ ^14^ ^]^ For the purpose of engineering a shrink‐resistant hydrogel, the aim is to combine two networks that are interpenetrating yet independent, whereby one provides rigidity and stability, preventing shrinkage, and the other is ductile and highly cytocompatible, mimicking the ECM. Currently, there is no cell‐laden IPN hydrogel available that has been shown to be resistant to fibroblast‐mediated shrinkage (Table S1 and S2, Supporting Information).

While collagen‐I is an excellent candidate for a ductile and highly cytocompatible material mimicking the ECM, silk is an excellent material to provide rigidity and stability. Silk from various sources, such as silkworm and spiders, has been shown to be a highly versatile natural biopolymer for tissue engineering, as it is cytocompatible, provides low immunogenicity, and can be processed into various forms such as films, foams, nanofibers, and hydrogels.^[^ ^15^ ^]^ Native silk‐based IPNs have previously been described with secondary networks comprising collagen‐I,^[^ ^16^ ^]^ fibrin,^[^ ^17^ ^]^ or methacrylated hyaluronic acid.^[^ ^18^ ^]^ Notably, only Buitrago et al. studied the effect on shrinkage. They have reported that hydrogels based on *Bombyx mori* silk fibroin/rat tail collagen‐I are resistant to compaction when human mesenchymal stem cells (hMSCs) from bone marrow (1.0 × 10^6^ per mL) are seeded onto the hydrogels.^[^ ^16^ ^]^ Importantly, hMSCs encapsulated in these *Bombyx mori* silk fibroin/rat tail collagen‐I hydrogels were not able to properly spread and proliferate.^[^ ^16^ ^]^

In this work, we have tested the hypothesis that IPNs based on nanofibrils self‐assembled from recombinant spider silk proteins,^[^ ^19^ ^]^ the primary network providing rigidity and stability, and rat tail collagen‐I, as ductile secondary network, prevent fibroblast‐mediated shrinkage but still promote complex cellular functions such as myoblast differentiation into skeletal myotubes and cardiomyocyte contractions (Figure 1B). Genetically engineered and recombinantly produced spider silk proteins are non‐cytotoxic and non‐immunogenic, and can be produced at a large scale with high purity and consistent quality.^[^ ^20^ ^]^ Additionally, possible modifications to the primary sequence, for example, addition of cell‐specific binding tags such as the sequence Arginine‐Glycine‐Aspartate (RGD), allow to tailor the silk to specific needs and applications providing high versatility.^[^ ^21^ ^]^

Our data demonstrate that 10 mg mL^−1^ eADF4(C16)‐RGD/1.6 mg mL^−1^ collagen‐I composite hydrogels allow cell adhesion and spreading, are resistant to cell‐mediated shrinkage and allow myotube formation as well as long‐term culture of engineered cardiac tissues exhibiting spontaneous contractions and drug responsiveness.

## 2 Results and Discussion

### 2.1 Composite Hydrogels of eADF4(C16)‐RGD and Collagen‐I Exhibit Synergistic Mechanical Properties

**Figure 2:**
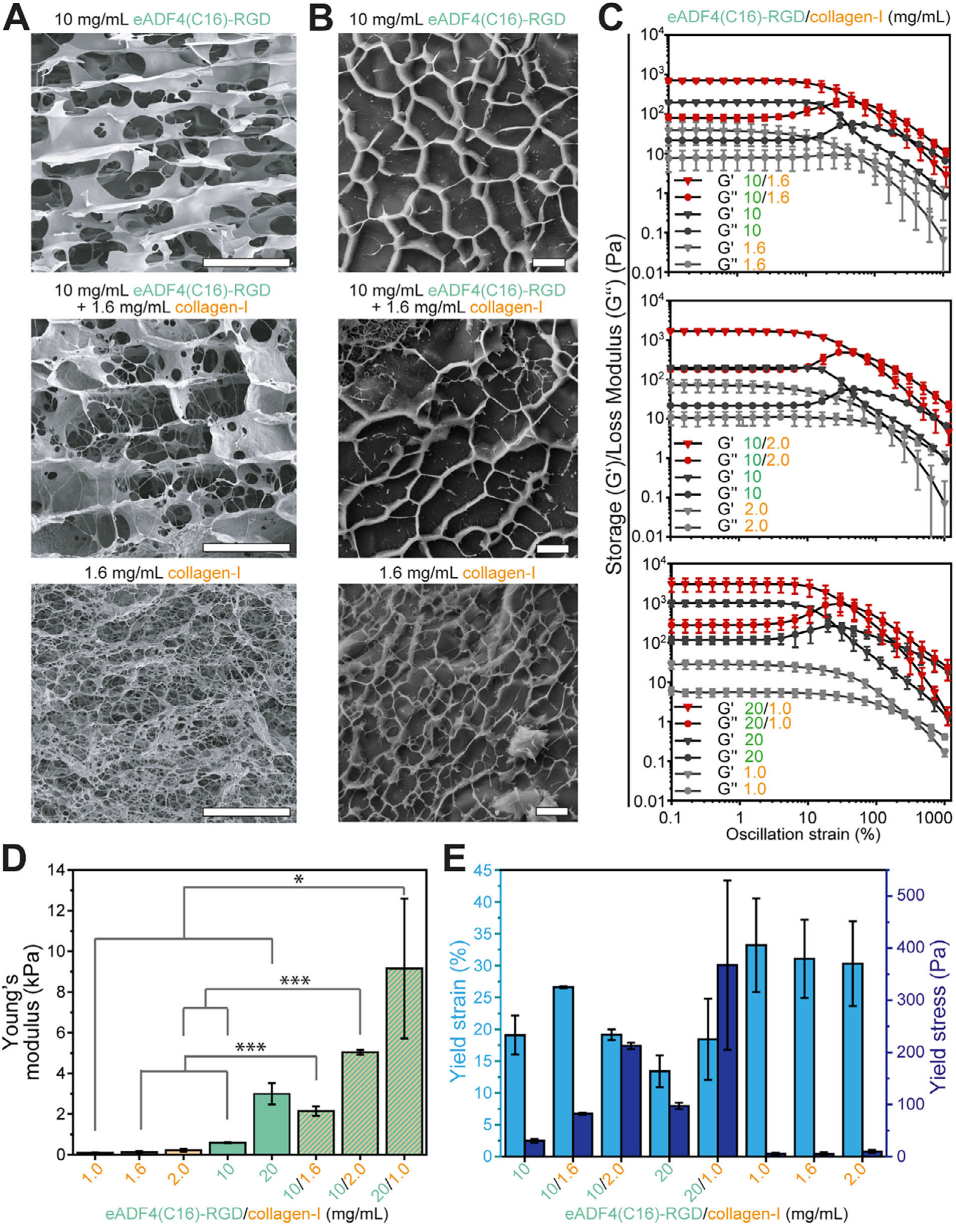
Properties of hydrogels made from eADF4(C16)‐RGD and collagen‐I. A) SEM images of recombinant silk and composite hydrogels. Scale bar: 20 µm. B) Cryo‐SEM images of gels at fracture sites showing plain or composite hydrogels as indicated. Scale bar: 4 µm. C‐E) Storage and loss moduli as function of strain obtained from amplitude sweep experiments (C) from which the Young's modulus E (D) and the yield strain and yield stress (E) were deduced (n = 3–5). Data: mean ± SD. *p < 0.05, ***p < 0.001. Statistics: one‐way ANOVA followed by Tukey Test to compare means.

The aim of this study was to fabricate an interpenetrating network (IPN) based on recombinant eADF4(C16)‐RGD and rat tail collagen‐I that allows cell encapsulation, proper cell function, and prevents cell‐mediated shrinkage. The recombinant spider silk protein eADF4(C16)‐RGD comprises sixteen repeats of a highly conserved sequence motif (named C) found in the core domain of *A. diadematus* dragline silk fibroin 4 (ADF4), tagged with an RGD‐motif.^[^ ^19^ ^]^ To ensure that the nucleation of self‐assembly was not affected by the hydrogel's composition, eADF4(C16)‐RGD and collagen‐I solutions were preassembled prior to mixing followed by the final assembly into the IPN. Scanning electron microscopy (SEM) analysis revealed that the composite hydrogels (10 mg mL^−1^ eADF4(C16)‐RGD/1.6 mg mL^−1^ collagen‐I) exhibited on one hand a honeycomb‐like structure typical for eADF4(C16)‐RGD and on the other hand fibrillar hydrogel network structures typical for collagen‐I, apparently lining the interior of the pores formed by spider silk (**Figure** **2**A). Notably, Cryo‐SEM was used to compare the overall pore structure of spider silk, collagen‐I, and the composite hydrogels showing that the pore size in composite hydrogels resembled the structure of pure eADF4(C16)‐RGD hydrogels and was markedly larger than the pore size observed in collagen‐I hydrogels (Figure 2B).

These data suggest that IPNs were formed in the composite hydrogel, in which eADF4(C16)‐RGD might play a dominant role in building the structural framework (honeycomb‐like structure) possibly providing rigidity and stability, while collagen‐I formed fibrillar structures known to provide appropriate physical and chemical cues for cytocompatibility.

As mechanics of hydrogels are critical for cell behavior in 3D cell culture,^[^ ^22^ ^]^ we next investigated the viscoelastic properties of hydrogels obtained from eADF4(C16)‐RGD and collagen‐I, utilizing a variety of combinations of concentrations. Previously, we have shown that fibroblasts can spread and proliferate in hydrogels made of 20 mg mL^−1^ eADF4(C16)‐RGD.^[^ ^23^ ^]^ For primary neonatal rat‐ and hiPSC‐cardiomyocytes, we have shown that hydrogels made of ≈3.5 mg mL^−1^ collagen‐I are suitable at cell densities of 2.5 × 10^7^ cells mL^−1^.^[^ ^24^ ^]^ Therefore, we produced hydrogels composed of 10 or 20 mg mL^−1^ eADF4(C16)‐RGD and 1.0, 1.6, or 2.0 mg mL^−1^ collagen‐I. Amplitude sweep experiments were conducted to determine the elastic modulus within the linear viscoelastic region (LVR), which was defined as the strain up to which the storage modulus decreased by 5% following ISO 6721‐10/ EN/DIN EN 14 770 norm (Figure 2C).

Elastic moduli of collagen‐I hydrogels matched the stiffness range reported by others (concentration, elastic modulus: 1.0 mg mL^−1^, 0.084 ± 0.02 kPa; 1.6 mg mL^−1^, 0.116 ± 0.067 kPa; 2.0 mg mL^−1^, 0.213 ± 0.074 kPa; Figure 2D).^[^ ^16^, ^25^ ^]^ Similarly, also the values for eADF4(C16)‐RGD hydrogels (10 mg mL^−1^, 0.595 ± 0.021 kPa; 20 mg mL^−1^, 2.996 ± 0.527 kPa; Figure 2D) were in full agreement with previous reports.^[^ ^23^ ^]^ The analysis of eADF4(C16)‐RGD/collagen‐I composite hydrogels revealed that the elastic moduli of all composite hydrogels were significantly higher compared to that of their individual components (10 mg mL^−1^ eADF4(C16)‐RGD + 1.6 mg mL^−1^ collagen‐I: 2.141 ± 0.232 kPa (3.6‐times higher), + 2.0 mg mL^−1^ collagen‐I: 5.039 ± 0.122 kPa (8.5 times higher); 20 mg mL^−1^ eADF4(C16)‐RGD + 1.0 mg mL^−1^ collagen‐I: 9.160 ± 3.440 kPa (3‐times higher), Figure 2D). Taken together, in all composite formulations tested, the elastic modulus was significantly higher than the sum of the elastic moduli of the respective individual components, indicating a synergistic relation of eADF4(C16)‐RGD and collagen‐I when forming a composite gel and further supporting the formation of an IPN.

When studying the loss modulus (G″) beyond the LVR, eADF4(C16)‐RGD showed an increase which represents an increased loss of stored energy through heat dissipation during structural breakage (e.g., microcracks) prior to pseudoplastic deformation (Figure 2C). This is a typical characteristic of brittle native^[^ ^26^ ^]^ and recombinant^[^ ^27^ ^]^ silk hydrogels. In contrast, collagen‐I hydrogels tend to show more elongated curves without an increase in loss modulus, which can be attributed to collagen fibers first aligning parallel to the direction of shear^[^ ^28^ ^]^ after which they slide past each other resulting in a broader shoulder and smoother drop in storage and loss modulus. Analysis of the composite hydrogels revealed that the storage‐to‐loss‐modulus relation is similar to that of plain recombinant eADF4(C16)‐RGD hydrogels, suggesting silk to be the predominant component determining the composite hydrogel's viscoelastic behavior as it is also present in a higher mass ratio compared to the collagen‐I constituent. These data substantiate the assumption that eADF4(C16)‐RGD provides rigidity and stability to the composite hydrogel.

To characterize the nature of the synergistic relation of eADF4(C16)‐RGD and collagen‐I, yield strains and yield stresses of the hydrogels were assessed using the tangent analysis method (Figure 2E). While addition of collagen‐I (1.0 and 1.6 mg mL^−1^) could increase the yield strain of eADF4(C16)‐RGD hydrogels (10 and 20 mg mL^−1^), which is a sign of improved elasticity, the addition of more collagen‐I (2.0 mg mL^−1^) did not result in any further improvement of yield strain and, thus, elasticity (Figure 2E). Consequently, there is an optimum ratio of eADF4(C16)‐RGD and collagen‐I, which is one characteristic of IPNs.^[^ ^29^ ^]^

Taken together, our data show that the combination of eADF4(C16)‐RGD and collagen‐I yields hydrogels with synergistic characteristics and tunable elasticity. However, at higher eADF4(C16)‐RGD concentrations (20 mg mL^−1^), hydrogel formation was less reproducible, as indicated by error bars relatively larger than for the other groups (Figure 2D,E). Notably, these composite hydrogels can be tuned to exhibit high matrix stiffness without the requirement of high polymer concentrations (e.g., 30 mg mL^−1^ eADF4(C16)‐RGD^[^ ^23^, ^27^ ^]^ or 70 mg mL^−1^ eADF4(C16)^[^ ^30^ ^]^) or chemical cross‐linking (30 mg mL^−1^ eADF4(C16)^[^ ^30^ ^]^), which both are known to potentially hamper cellular functions.

### 2.2 eADF4(C16)‐RGD Prevents Fibroblast‐Mediated Collagen‐I Hydrogel Shrinkage

**Figure 3:**
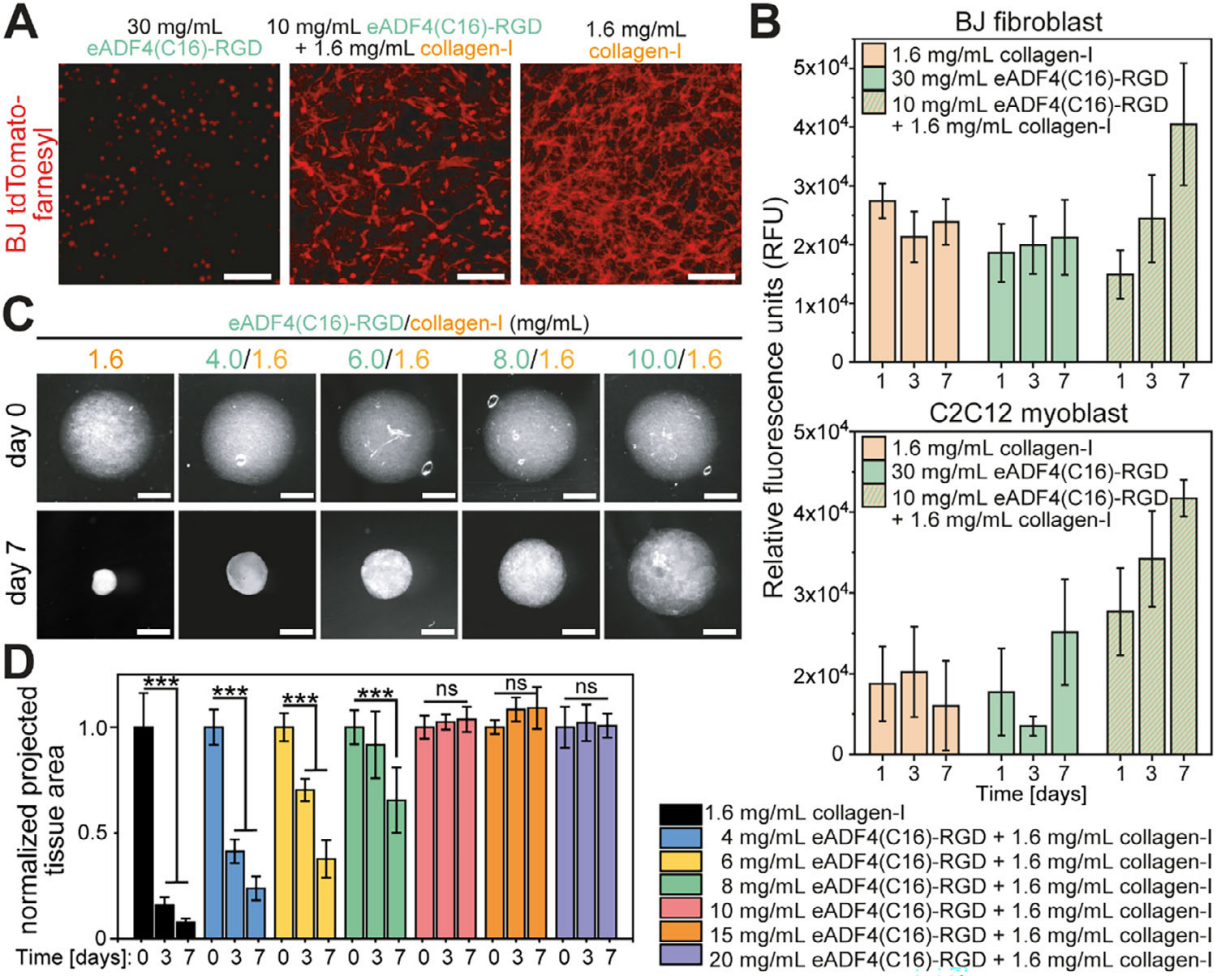
Hydrogels consisting of 10 mg/mL eADF4(C16)‐RGD/ 1.6 mg mL^−1^ collagen‐I Spider silk/collagen‐I are shrink‐resistant. A) Z‐projection summed slice images of BJ tdTomato‐farnesyl reporter cells cultured for 7 days in the indicated hydrogels. Scale bar: 100 µm. B) Quantitative analysis of the metabolic activity of BJ fibroblasts and C2C12 cells in the indicated hydrogels assessed with CellTiter‐Blue®. Data: mean ± SD. n = 15–16. C) Representative examples of 25 µL hydrogels containing 1.0 × 10^6^ Mio mL^−1^ BJ fibroblasts before and 7 days after culture on non‐adhesive plates. Scale bar: 2 mm. D) Quantitative analysis of compaction. Data: mean ± SD. n = 9. ns: not significant; ***p < 0.001. Statistics: two‐way ANOVA followed by Tukey Test to compare means.

Fibroblasts are essential for the optimal function of tissues^[^ ^12^ ^]^ but are also the most active cells in regards of ECM remodeling.^[^ ^12^, ^13^ ^]^ This is a significant problem, as most of the currently used materials in tissue engineering are remodeled by fibroblasts resulting in cell‐induced construct shrinkage and, thus, in a very poor shape fidelity, considering that shape is very important for function.^[^ ^1^ ^]^ First, we tested whether eADF4(C16)‐RGD/collagen‐I composite hydrogels were suitable for fibroblast adhesion and survival. For this purpose, BJ tdTomato‐farnesyl reporter cells^[^ ^31^ ^]^ (1.0 × 10^6^ Mio mL^−1^) were encapsulated in 30 mg mL^−1^ eADF4(C16)‐RGD (allows rapid gelation for cell encapsulation and is well characterized in the literature),^[^ ^27^, ^32^ ^]^ 1.6 mg mL^−1^ collagen‐I (based on the yield stress data in Figure 2E) or composite hydrogels containing 10 mg mL^−1^ eADF4(C16)‐RGD and 1.6 mg mL^−1^ collagen‐I (based on data in Figure 2E), cultured for 7 days, and analyzed using fluorescence microscopy (**Figure** **3**A).

The farnesylated fluorescent protein (tdTomato) localizes to the cell membrane, enabling the evaluation of cell morphology. These data showed that BJ fibroblasts could adhere and spread, exhibiting cytoplasm extensions indicative for filopodia in collagen‐I and composite hydrogels but not in 30 mg mL^−1^ eADF4(C16)‐RGD hydrogels (round cells). These data substantiate our conclusion that collagen‐I offers in the composite hydrogel appropriate physical and chemical cues for cytocompatibility. Notably, the cell density in 1.6 mg mL^−1^ collagen‐I hydrogels was markedly higher than in composite hydrogels. This can be either explained by increased cytotoxicity of the composite hydrogel, a higher proliferation rate in the collagen‐I hydrogels, or a markedly higher compaction rate of collagen‐I hydrogels compared to composite hydrogels. To determine whether the hydrogels exhibit different cytotoxic or proliferative effects, BJ fibroblasts (1.0 × 10^6^ Mio mL^−1^) and C2C12 skeletal myoblasts (2.0 × 10^6^ Mio mL^−1^) were embedded in the different hydrogels and analyzed using the CellTiter‐Blue cell viability assay (Promega) at day 1, 3, and 7 (Figure 3B). This assay utilizes the capacity of living cells to reduce the indicator dye resazurin into resorufin, which is highly fluorescent. Notably, under most experimental conditions, there is a linear relationship between the fluorescent signal and the number of viable cells. Our data revealed that the fluorescence intensity in 1.6 mg mL^−1^collagen‐I hydrogels remained relatively constant from day 1 to day 7. This indicates that the number of viable cells remained constant and suggests that the increased cell density in collagen‐I hydrogels (Figure 3A) was not due to an increase in cell number but caused by the shrinkage of the hydrogel. In contrast, the fluorescence intensity in 10 mg mL^−1^ eADF4(C16)‐RGD/1.6 mg mL^−1^ collagen‐I composite hydrogels increased markedly from day 1 to day 7 suggesting that there are more cells, meaning the cells can proliferate in the composite hydrogels. One explanation could be that the shrink‐resistance of the composite hydrogels provides cells with more space allowing proliferation.

In order to validate that the addition of eADF4(C16)‐RGD protected collagen‐I hydrogels from cell‐mediated compaction, BJ fibroblasts (1.0 × 10^6^ Mio mL^−1^) were encapsulated in a variety of composite hydrogels and cultivated on non‐adhesive culture plates as detached 25 µL droplets (Figure 3C). The size of the droplets was monitored and quantified based on darkfield images taken at 0, 3, and 7 days of culture (Figure 3D).

Based on the elastic modulus and yield stress data in Figure 2E, 1.6 mg mL^−1^ collagen‐I was identified as an optimal concentration for the composite hydrogels. Based thereon, we tested eADF4(C16)‐RGD/collagen‐I composite hydrogels with varying recombinant silk concentrations from 4 to 20 mg mL^−1^ and 1.6 mg mL^−1^ collagen‐I. As previously described,^[^ ^33^ ^]^ collagen‐I hydrogels were rapidly compacted to 16% ± 4% and 8% ± 2% at day 3 and day 7 of their original area at day 0, respectively (Figure 3D). The rate of compaction was significantly reduced in the presence of increasing amounts of eADF4(C16)‐RGD. At a concentration of 10 mg mL^−1^ eADF4(C16)‐RGD or higher, shrinkage was completely prevented. Notably, 10 mg mL^−1^ eADF4(C16)‐RGD/1.6 mg mL^−1^ collagen‐I composite hydrogels were also shrink‐resistant in the presence of NIH/3T3 fibroblasts as well as C2C12 myoblasts (Figure S1, Supporting Information).

In summary, our data demonstrated that hydrogels made of IPNs of 10 mg mL^−1^ eADF4(C16)‐RGD/1.6 mg mL^−1^ collagen‐I exhibit highly reproducible mechanical properties (Figure 2) and are shrink‐resistant (Figure 3).

### 2.3 eADF4(C16)‐RGD/Collagen‐I Composite Hydrogels Allow Skeletal Myotube Formation

**Figure 4:**
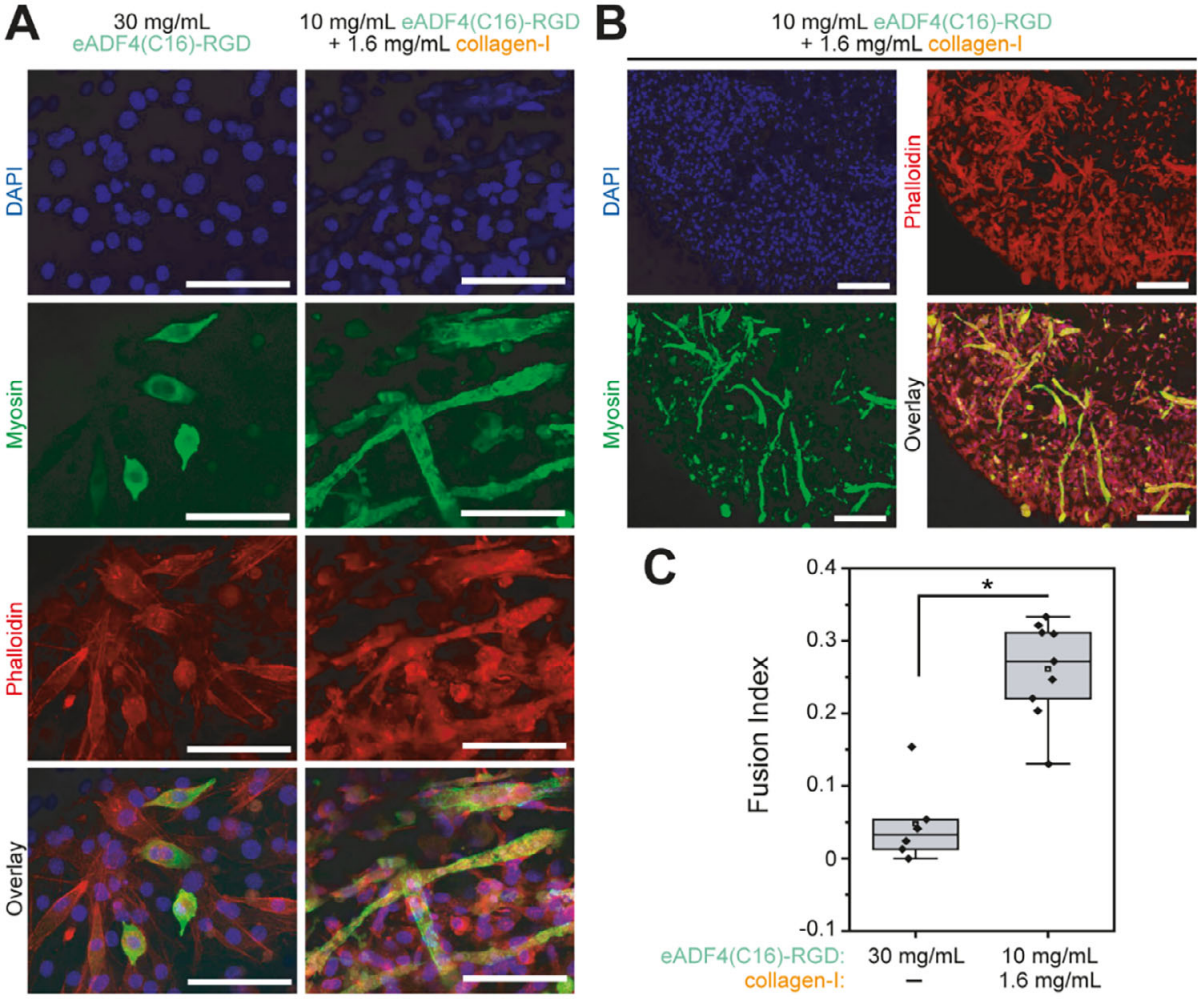
C2C12 cells formed skeletal myotubes in eADF4(C16)‐RGD/collagen‐I composite hydrogels. A, B) Maximum projection images of C2C12 myoblasts cultured in differentiation medium for 7 days in the indicated hydrogels stained for myosin (green, marker of skeletal muscle differentiation), F‐actin (phalloidin, cytoskeletal marker), and DNA (DAPI. Nuclei). Scale bars: A = 100 µm, B = 100 µm. C) Box plot showing quantitative analysis of myotube formation in A and B. Data shows upper and lower quartile (0.25 – 0.75) with whiskers indicating 1.5 interquartile range value. n = 5–9. *p < 0.05. Statistics: Students t‐test.

To determine if the shrink‐resistant 10 mg mL^−1^ eADF4(C16)‐RGD/1.6 mg mL^−1^ collagen‐I hydrogels supported more complex cellular functions and were superior to non‐shrinkable plain eADF4(C16)‐RGD hydrogels, C2C12 skeletal myoblasts were embedded into 30 mg mL^−1^ eADF4(C16)‐RGD as well as the 10 mg mL^−1^ eADF4(C16)‐RGD/1.6 mg mL^−1^ collagen‐I composite hydrogels. Note, mechanical properties of 10 mg mL^−1^ eADF4(C16)‐RGD/1.6 mg mL^−1^ collagen‐I hydrogels are similar to the mechanical properties of other materials (matrix stiffness in the lower kPa range) previously shown to be suitable for culturing skeletal and cardiac muscle cells.^[^ ^34^ ^]^ Embedded cells were first cultured in growth media for 7 days and subsequently subjected to a differentiation medium containing horse serum (2%) for another 5 days to determine if the hydrogels allowed the differentiation and fusion of the C2C12 myoblasts into myotubes (**Figure** **4**).

Differentiated C2C12 cells were identified by anti‐myosin staining, and the formation of myotubes was assessed based on morphological features (Figure 4A,B) and quantified (Figure 4C). This analysis revealed that C2C12 myoblasts in 30 mg mL^−1^ eADF4(C16)‐RGD hydrogels barely differentiated and rarely formed myotubes (Figure 4A). In contrast, C2C12 cells in the composite hydrogels readily differentiated and formed large, elongated myotubes (Figure 4A) throughout the entire gel (Figure 4B). These data indicated that the 10 mg mL^−1^ eADF4(C16)‐RGD/1.6 mg mL^−1^ collagen‐I composite hydrogels not only allowed cells to adhere and prevent fibroblast‐mediated shrinkage, but to even differentiate and fuse.

### 2.4 eADF4(C16)‐RGD/Collagen‐I Hydrogels Are Suitable for Cardiac Tissue Engineering

Previously, we have shown that materials made of silk proteins are very promising candidates for cardiac tissue engineering.^[^ ^21^, ^35^ ^]^ Yet, our approach was so far limited to either acellular scaffolds that needed to be seeded post‐fabrication, which prevented the generation of highly functional tissues,^[^ ^36^ ^]^ or to 2D or coating applications. In addition, we have shown that collagen‐I‐based hydrogels are well suited for cardiac tissue engineering.^[^ ^24^, ^37^ ^]^ However, these engineered tissues consisted only of hiPSC‐cardiomyocytes, as the addition of fibroblasts, known to greatly improve the contractility of engineered cardiac tissues,^[^ ^38^ ^]^ would have compacted the tissues resulting in the loss of any fabrication design. Considering that heart disease is the leading cause of death worldwide causing an enormous socioeconomic burden,^[^ ^39^ ^]^ we wondered if 10 mg mL^−1^ eADF4(C16)‐RGD/1.6 mg mL^−1^ collagen‐I hydrogels are suitable for cardiac tissue engineering, as engineered cardiac tissues are valuable tools to model disease and study drug response. In addition, pre‐clinical animal studies suggested that transplantation of such engineered tissues can even improve heart function after infarction.^[^ ^40^ ^]^

Notably, the mechanical properties of a matrix are essential for the proper behavior of cells, such as maturation and contractility. It has been reported that a matrix stiffness of 11–17 kPa is well suited for culturing embryonic chick cardiomyocytes^[^ ^41^ ^]^ and 1 kPa for human embryonic cardiomyocytes.^[^ ^34a^ ^]^ hiPSC‐cardiomyocytes currently present the most promising source of cells for cardiac tissue engineering for pre‐clinical testing or clinical translation, even though they exhibit embryonic characteristics.^[^ ^42^ ^]^ Thus, the 10 mg mL^−1^ eADF4(C16)‐RGD/1.6 mg mL^−1^ collagen‐I composite hydrogel with an elastic modulus of ≈2 kPa (Figure 2E) turned out to be a promising material for cardiac tissue engineering and was chosen for subsequent experiments. We encapsulated hiPSC‐cardiomyocytes in composite hydrogels or hydrogel controls at 25 × 10^6^ cells mL^−1^. Over a cultivation period of 14 days, we observed a compaction of 1.6 mg mL^−1^ collagen‐I hydrogels yielding a shrinkage of ≈50% of their initial size similar to that of fibroblast‐laden hydrogels (Figure S2, Supporting Information). In contrast, composite hydrogels showed only a ≈10% reduction in size, while plain 30 mg mL^−1^ eADF4(C16)‐RGD hydrogels did not show any obvious compaction.

**Figure 5:**
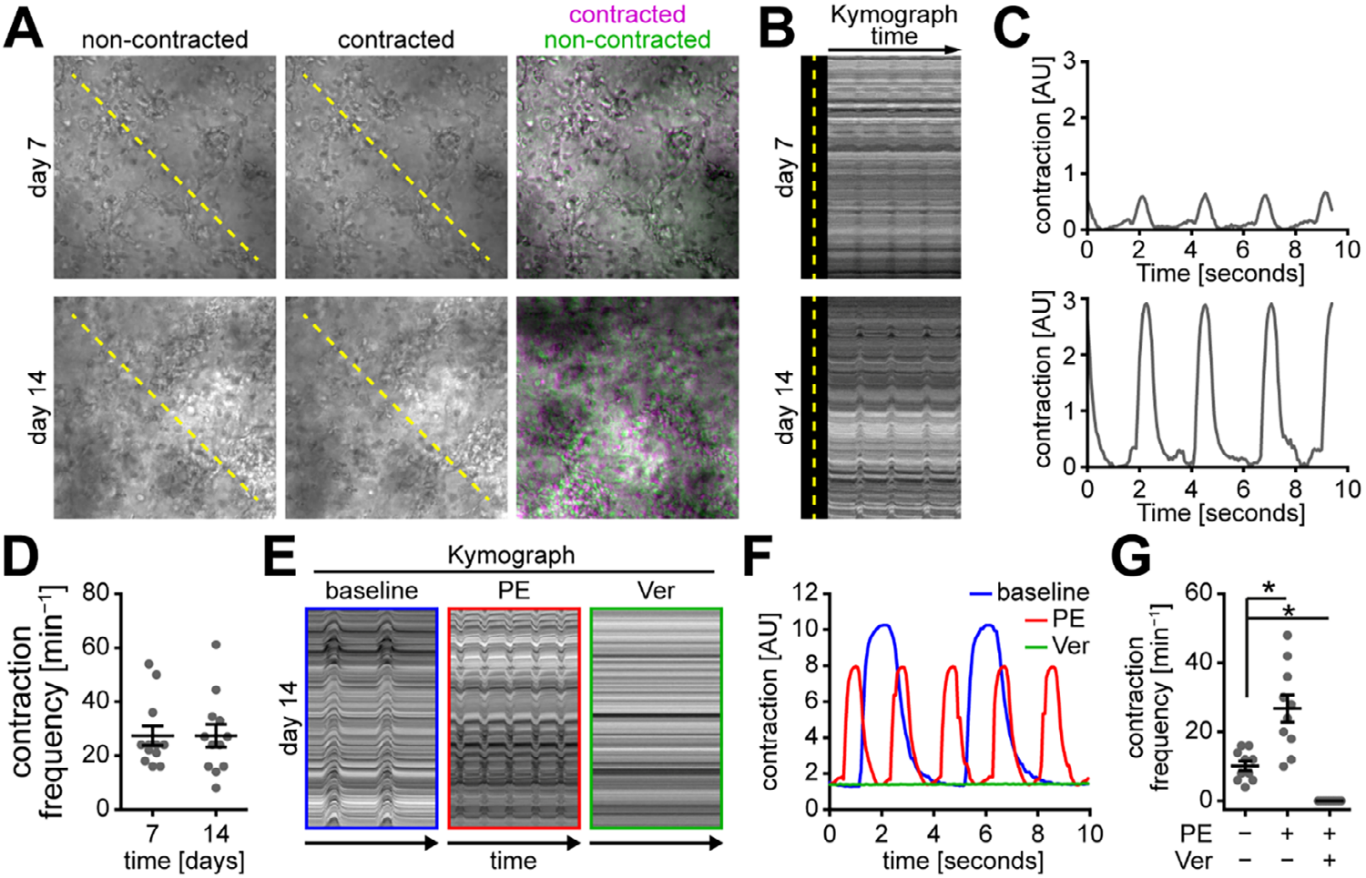
eADF4(C16)‐RGD/collagen‐I hydrogels are suitable for cardiac tissue engineering. A) Individual or merged false‐colored darkfield images of contracted (magenta) and non‐contracted (green) tissues (25 × 10^6^ hiPSC‐cardiomyocytes mL^−1^ embedded in 10 mg mL^−1^ eADF4(C16)‐RGD/1.6 mg mL^−1^ collagen‐I hydrogel) on day 7 and day 14 post‐fabrication, illustrating deflection during contraction. B) Kymograph analyses along the yellow dashed lines indicated in A. C) Corresponding MUSCLEMOTION analyses of tissues in A. D) Contraction frequency (n  =  12). Data: mean ± SEM. E) Representative kymograph analyses of tissues (day 14) at baseline and after treatment with phenylephrine (PE, 50 µM) and verapamil (Ver, 1 µM). F) MUSCLEMOTION analyses of tissues referred to in E. G) Contraction frequency of hybrid hydrogel tissue constructs (day 14) at baseline and after PE and Ver treatment (n  =  10). Data: mean ± SEM. *p < 0.05. ANOVA followed by post‐hoc analysis according to Dunnett.

Currently, it is unclear if compaction is required for the function of cardiac tissues, and previous reports of collagen‐based engineered cardiac tissues argued on compaction or condensation being a prerequisite for proper contractility.^[^ ^38^ ^]^ Notably, strong spontaneous contractions of 10 mg mL^−1^ eADF4(C16)‐RGD/ 1.6 mg mL^−1^ collagen‐I composite hydrogel constructs were observed within the first week of cultivation (**Figure** **5**A‐C; Movie S1, Supporting Information).

These contractions became more prominent with ongoing cultivation until day 14, as apparent in more pronounced peaks in kymograph‐based analyses (Figure 5B; Movie S2, Supporting Information) and greater contraction amplitudes (Figure 5C) calculated using MUSCLEMOTION analysis.^[^ ^43^ ^]^ Contraction frequency, however, remained constant throughout this period at ≈27.4 min^−1^ (Figure 5D). These data suggest that hydrogel compaction is not required to engineer functional cardiac tissues.

To serve as suitable models for drug testing and mechanistical studies or transplants, engineered cardiac constructs must demonstrate responsivity/sensitivity to pharmacological stimulation. We thus treated composite hydrogel‐based cardiac constructs on day 14 post‐fabrication with phenylephrine (PE), an adrenergic agonist, followed by incubation with verapamil (Ver), an inhibitor of calcium channels (Figure 5E‐G; Movie S3, Supporting Information). Tissue constructs showed a significant ≈2.6‐fold increase in beating frequency upon PE‐stimulation, while the addition of Ver caused spontaneous contractions to stop.

### 2.5 Cardiomyocytes Exhibit a Well‐Organized Sarcomeric Apparatus and Electrical Coupling in eADF4(C16)‐RGD/Collagen‐I Hydrogels

**Figure 6:**
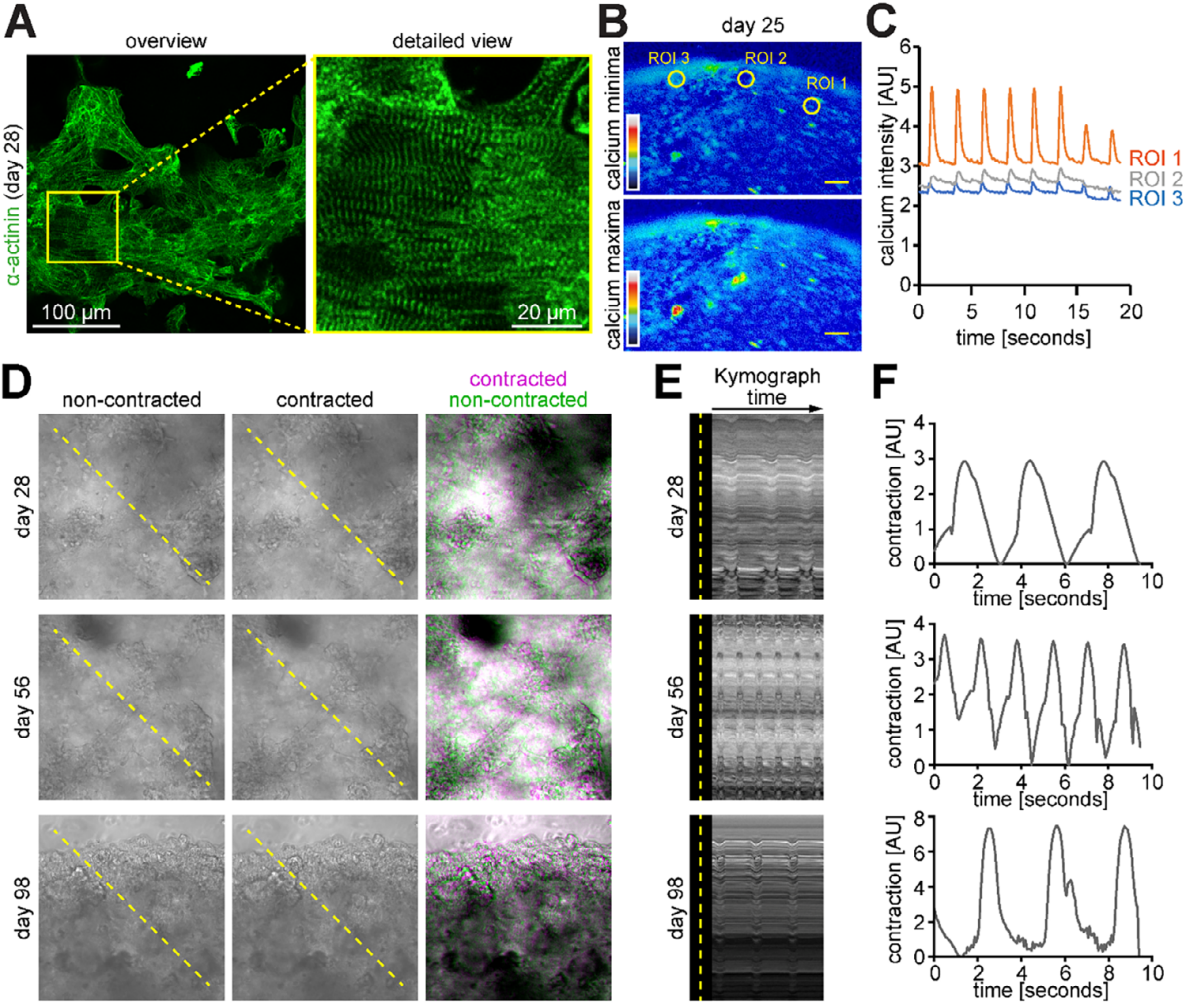
Long‐term cultivation of cardiac 10 mg mL^−1^ eADF4(C16)‐RGD/1.6 mg mL^−1^ collagen‐I constructs. A) Representative maximum intensity projections of confocal z‐stacks of cardiac constructs on day 28, stained for sarcomeric alpha‐actinin. B, C) Local calcium minima and maxima concentration based on Fluo‐4 calcium analysis showing representative examples of intracellular calcium changes of cardiomyocytes in 3D engineered tissues for different regions of interests (ROIs). (B) Analyzed ROI are indicated by yellow circles. The chart of color range represents minimum and maximum signals from purple to red, respectively. Scale bars: yellow: 20 µm. C) Quantified fluorescent signal shown for individual ROIs based on the analyzed videos exemplified in (B). D) Individual or merged false‐colored darkfield images of contracted (magenta) and non‐contracted (green) states of tissue constructs comprising 25 × 10^6^ cells mL^−1^ hiPSC‐cardiomyocytes embedded in 10 mg mL^−1^ eADF4(C16)‐RGD/1.6 mg mL^−1^ collagen‐I on days 28, 56, and 98, illustrating deflection during contraction. E) Kymograph analyses along the yellow dashed lines indicated in (D). F) Corresponding MUSCLEMOTION analyses of tissue constructs depicted in (D).

In order to generate maximal force, it is crucial that cardiomyocytes contain a well‐organized sarcomeric apparatus and that cardiomyocytes exhibit proper electrical coupling within the engineered tissues. Thus, we have investigated the contractile apparatus of hiPSC‐cardiomyocytes within engineered tissues by immunostaining sarcomeric alpha‐actinin. These data showed that hiPSC‐cardiomyocytes within the 10 mg mL^−1^ eADF4(C16)‐RGD/1.6 mg mL^−1^ collagen‐I composite hydrogel constructs exhibited a highly organized and aligned sarcomeric apparatus (**Figure** **6**A). Next, we investigated the calcium handling of hiPSC‐cardiomyocytes within eADF4(C16)‐RGD/ collagen‐I composite hydrogels analyzing live videos of Fluo‐4 calcium‐labelled engineered cardiac tissues (Figure 6B,C; Movie S4, Supporting Information). We detected synchronous calcium flux peaks. These data indicate that hiPSC‐cardiomyocytes within eADF4(C16)‐RGD/ collagen‐I composite hydrogels efficiently coupled and exhibited enhanced calcium handling behavior, concurrent with the regular beating observed in all constructs at the different investigated time points (Figures 5 and 6; Movies S1–S8, Supporting Information). Notably, composite hydrogel‐based cardiac constructs continued to display spontaneous contractions for up to 98 days (14 weeks) in culture without the need for external stimulation (Figure 6D‐F; Movies S5–S8, Supporting Information).

Collectively, 10 mg mL^−1^ eADF4(C16)‐RGD/1.6 mg mL^−1^ collagen‐I hydrogels are shrink‐resistant and allow the engineering of long‐term stable, functional human heart tissues exhibiting physiological responses to PE and Ver treatment.

## 3 Conclusion

We conclude that we have developed a novel cytocompatible, shrink‐resistant hydrogel with tunable properties, which allows complex cellular behaviors including cell attachment and spreading, cell fusion, and cell contraction. Several lines of evidence support this conclusion. Rheological analyses revealed that the elastic modulus was significantly higher in the composite hydrogels than the sum of the elastic moduli of the respective individual components eADF4(C16)‐RGD and collagen‐I. Composite hydrogels consisting of 10 mg mL^−1^ eADF4(C16)‐RGD and 1.6 mg mL^−1^ collagen‐I were shrink‐resistant to several different cell‐types. C2C12 myoblasts differentiated and fused to skeletal myotubes in the composite hydrogels. Finally, hiPSC‐cardiomyocytes embedded in this composite hydrogel exhibited well‐organized sarcomeres as well as spontaneous and synchronous contractions that persisted for several months. These contractions could be modulated in frequency by pharmacological stimulation. Thus, the here developed hydrogel represents a new material suitable for the engineering of a wide range of tissues.

While there are still challenges to overcome, the here developed material conferring high shape fidelity opens up new possibilities for generating complex functional tissues. In future studies, it will be important to utilize and optimize eADF4(C16)‐RGD/collagen‐I composite hydrogels for specific applications, e.g. in combination with additives to enhance tissue performance, and to determine its shrink‐resistance and applicability in vivo. Notably, in this context our previous data indicate that eADF4(C16)‐RGD hydrogels in vivo are biocompatible, promote vascularization and do not elicit an immune response.^[^ ^32^ ^]^

## 4 Experimental Section

### Hydrogel Preparation

Acid soluble rat tail telo‐collagen‐I hydrogels were produced using a commercial kit (AdvancedBioMatrix, stock concentration of 4.0 mg mL^−1^) and were prepared as by the manufacturer's instructions using a neutralization solution to adjust optimal pH and salt concentration. The production, purification and processing of eADF4(C16)‐RGD hydrogels is described elsewhere.^[^ ^19^ ^]^ Briefly, the lyophilized protein was denatured using 6 M guanidinium thiocyanate (Carl Roth GmbH) at a concentration of 15 mg mL^−1^ for 60 min at room temperature using an overhead shaker. The silk solution was then filtered using 0.2 µm non‐pyrogenic syringe filters (Sarstedt) to remove undissolved remnants and dialyzed against an excess of 10 mM Tris/HCl buffer (pH 7.5) at 4 °C using tubes with a cut‐off range of 6–8 kDa (Spectra/Por, Repligen) over a period of 16 h. To concentrate the silk solution for further processing, it was dialyzed against 25 wt.% PEG (40 000 Da; VWR). The concentrated silk solution was then collected, centrifuged for 20 min at 17 000 x g, and the protein concentration was determined using a ND‐100 UV/Vis spectrophotometer (Nano‐drop Technologies Inc.). eADF4(C16)‐RGD/collagen‐I composite hydrogels were prepared by physical pre‐crosslinking the concentrated silk solution in a water bath at 37 °C for 2 h with an initial concentration of 35.3 mg mL^−1^ prior to mixing with neutralized collagen‐I at respective volumes. Note, the concentration of the collagen‐I stock limited the upper range for the collagen content when working with formulations using 20 mg mL^−1^ eADF4(C16)‐RGD. Higher initial eADF4(C16)‐RGD stock concentrations yields difficulties upon processing due to rapid self‐assembly. Concerning lower eADF4(C16)‐RGD concentrations, formulations with low concentrations such as 10 mg mL^−1^ require gelation times exceeding 24 h to form stable gels, which is not compatible with cells. In contrast, the presence of collagen‐I in composite gels accelerates the gelation markedly allowing cell encapsulation, even when using 10 mg mL^−1^ eADF4(C16)‐RGD. This observation is in agreement with previous studies using *Bombyx mori* silk.^[^ ^16^ ^]^ Therefore, 30 mg mL^−1^ eADF4(C16)‐RGD hydrogels were chosen as control group for plain silk gels as this concentration allowed rapid gelation for cell encapsulation and is well characterized in the literature. Gels were then incubated for 1 h at 37 °C and 5% atmospheric CO_2_ content in a cell culture incubator until fully assembled.

### Rheological Characterization

Viscoelastic gel properties were determined by oscillating amplitude sweep experiments on a strain‐controlled DHR‐2 rheometer (TA instruments) using a sand‐blasted 25 mm diameter upper plate geometry. Sweeps were carried out in a range of 0.1% – 1000% at a frequency of 10 rad s^−1^. Gels were cast into polystyrene cell culture plates punched out with a 26 mm hole puncher and transferred into the measuring gap (500 µm). Data analysis was performed using the TRIOS software (v5.1.1). The elastic modulus was calculated from Equation (1) with the Poisson ratio (v) estimated to be 0.5.^[^ ^44^ ^]^

(1) $$E=2G\;\left(1+v\right)$$

The yield stress and strain were determined using the Onset analysis tool in TRIOS.

### Scanning Electron Microscopy (SEM)

Gel samples were dehydrated using an ethanol series and a subsequent exchange with tert‐butanol (Carl Roth GmbH) prior to lyophilization as described previously.^[^ ^45^ ^]^ Briefly, gels were incubated with cold ethanol at increasing concentrations (30%, 50%, 70%, and 100%) for 5 min in each step followed by an exchange with 50% and 100% tert‐butanol in ethanol for 5 min on ice. Dehydrated samples were then lyophilized to remove the tert‐butanol, fractured, and attached to sample carriers using Leit‐C conductive carbon ink (Plano GmbH) and coated with 1.3 nm platinum using an EM ACE600 (Leica). Images were acquired on an ApreoVS SEM device (ThermoFisher Scientific) using an ETD detector and applying a beam current of 25 pA and an acceleration voltage of 2.00 kV.

### Cryo SEM

The hydrogel samples were rapidly frozen in slushed nitrogen at −210 °C after placing them between aluminum plates (d = 3 mm) with a 2 mm notch for sample fixation. All following transfer steps were performed at −140 °C using an EM VCT100 cryo‐shuttle (Leica Microsystems). To generate a freshly fractured hydrogel surface, one of the aluminum plates was knocked off and freeze etched for 15 min at −85 °C under high vacuum (< 1 × 10^3^ mbar) in a Sputter Coater machine (ACE 400, Leica Microsystems). Afterwards, samples were sputtered with 3 nm platinum and transferred to the SEM chamber (Crossbeam 340, Zeiss). Images of the hydrogel surface morphology were taken at −160 °C using an acceleration voltage of 8 kV.

### Cell Culture

Human induced pluripotent stem cells were cultured and differentiated as previously described.^[^ ^37a^ ^]^ In brief, hiPSC were cultured in StemMACS iPS brew XF (Miltenyi Biotec) on cell culture plates coated with Matrigel (Corning) or Geltrex (ThermoFisher Scientific). For cardiac differentiation, media were switched to differentiation media, comprising RPMI 1640 (ThermoFisher Scientific), supplemented with 2% B‐27 minus Insulin (ThermoFisher Scientific) and 100 µM ascorbic acid. For the first 24 h (day 0–1), differentiation medium was supplemented with 8–10 µM CHIR99021 (Hycultec). During days 3–5, differentiation medium was supplemented with 5 µM IWR1‐endo (Selleckchem). From day 7 on, differentiated cardiomyocytes were maintained in RPMI1640 supplemented with 2% B27 (ThermoFisher Scientific). Metabolic selection was performed during days 9–14, by switching to RPMI 1640 without glucose, supplemented with 5 mM sodium‐DL‐lactate and 100 µM ascorbic acid. Purified hiPSC‐cardiomyocytes were dissociated using Accutase (Sigma Aldrich) to prepare 3D gel‐drop cultures. The human BJ fibroblast cell line expressing tdTomato‐farnesyl (ATCC, CRL‐4001; BJ1‐hTERT, RRID: CVCL_6573) to visualize the plasma membrane of spreading cells in 3D hydrogel cultures (generated by Lena Fischer and Ingo Thievessen)^[^ ^31^ ^]^ and the parental cell line were generously provided by Prof. A. Bosserhoff (FAU Erlangen, Germany). BJ cells were cultured in high‐glucose EMEM (Gibco, ThermoFisher Scientific) supplemented with 10% bovine calf serum (Sigma‐Aldrich Chemie GmbH), 50 µg mL^−1^ gentamycin (Biowest) and additionally 1 µg mL^−1^ puromycin (Gibco, ThermoFisher Scientific) for selection of tdTomato‐farnesyl positive cells. Cells were sub‐cultured every other day at 70 – 80% confluency using 0.25% trypsin/EDTA (Gibco, ThermoFisher Scientific). The NIH3T3 fibroblast cells were cultured in high‐glucose DMEM (Gibco, ThermoFisher Scientific) supplemented with 10% bovine calf serum (Sigma‐Aldrich Chemie GmbH), 1% (v/v) GlutaMax (Gibco, ThermoFisher Scientific) and 100 U Penicillin/ 0.1 mg mL^−1^ Streptomycin (Gibco, ThermoFisher Scientific). The C2C12 murine myoblast cell line (ATCC, CRL‐1772) was cultured in high‐glucose DMEM (Gibco, ThermoFisher Scientific) growth media supplemented with 10% bovine calf serum (Sigma‐Aldrich Chemie GmbH), 20 mM HEPES, 1% (v/v) GlutaMax (Gibco, ThermoFisher Scientific) and 100 U Penicillin/ 0.1 mg mL^−1^ Streptomycin. To induce differentiation growth medium was exchanged with differentiation media containing high‐glucose DMEM (Gibco, ThermoFisher Scientific) supplemented with 2% horse serum, 20 mM HEPES, 1% (v/v) GlutaMax (Gibco, ThermoFisher Scientific), 1x Insulin‐Transferrin‐Selenium Supplement (Gibco, ThermoFisher Scientific) and 100 U Penicillin/ 0.1 mg mL^−1^ Streptomycin.

### Preparation of 3D Gel‐Drop Cultures

Cells were harvested by centrifugation of the respective volume to obtain the desired numbers of cells for 5 min at 300 x g. Excess media was aspirated and the cells resuspended in growth media to achieve a final media content of 15 vv.%. Respective volumes of the eADF4(C16)‐RGD stock solution (35.3 mg mL^−1^), neutralized collagen‐I solution (3.7 mg mL^−1^), sterile ultrapure water (MilliQ) and cells in suspension were mixed to obtain respective ink formulations with a final cell count of 25.0 × 10^6^ cells mL^−1^ for hiPSC‐cardiomyocytes or 2.0 × 10^6^ cells mL^−1^ for BJ cells. Individual droplets of 25 µL hydrogels were then placed into 8‐well chamber slides and incubated for 1 h at 37 °C (5% CO_2_) for gelation to occur, before adding culture medium. Tissues were cultured free floating, after having been dislodged from the bottom of the culture vessel by the addition of medium or using a small spatula.

### Hydrogel Compaction Assay

For determining the change in surface area of hydrogels, hiPSC‐cardiomyocytes (25.0 × 10^6^ cells mL^−1^), C2C12 myoblast (2.0 × 10^6^ cells mL^−1^), BJ fibroblast (1.0 × 10^6^ cells mL^−1^) and NIH3T3 fibroblast (1.0 × 10^6^ cells mL^−1^) cells were encapsulated in respective hydrogel formulations. Before gels were fully formed, individual droplets (25 µL) were placed into single wells of an untreated 48‐well tissue culture plate and incubated at 37 °C, 5% CO_2_ for 1 h. After gels had fully assembled, culture medium was added according to the cell line encapsulation. Droplets were detached and imaged using darkfield microscopy for better outline contrasting. The projected surface area of constructs was determined from darkfield images using ImageJ/FIJI. In brief, images were converted to 8‐bit gray scale images and gaussian blur filter applied with a sigma radius of 3. An auto intensity threshold (“Default dark”) and contrast enhancement (saturation = 0.35) was applied to the 8‐bit images and FIJI's “Analyze Particles” tool was utilized to identify the tissue construct outline and to measure its area.

### Cell Proliferation Assay

To confirm that cells were viable and grew within the 3D environment of silk/collagen‐I composite gels, the metabolic activity of BJ fibroblast and C2C12 mouse myoblasts cells was monitored using the commercial CellTiter‐Blue Viability Assay (Promega). The assay is based on the reduction of resazurin to the fluorescent active resorufin by aerobic respiration of metabolically active cells where the increase of resorufin is considered proportional to the number of in average equally, metabolically active cells. Silk/ collagen‐I composite hydrogel drops were prepared as described previously with an initial cell density of 2.0 × 10^6^ cells mL^−1^ for C2C12 and 1.0 × 10^6^ cells mL^−1^ for BJ per milliliter hydrogel. The assay was carried out on every other day starting at 24 h post‐embedding (days 1, 3, 5, 7, and 9). Hydrogel drops were washed twice with DPBS and then transferred to fresh 24‐well plates prior to carrying out the viability assay. Individual droplets were then incubated in Cell Titer Blue for 2.5 h at 37 °C prior to obtaining 100 µL of the supernatant which was transferred to an opaque 96‐well plate, and the amount of resorufin was detected using a Mithras LB 940 micro plate reader (Berthold Technologies) with Ex. 570 nm / Em. 600 nm filters. The hydrogel drops were subsequently washed twice with Dulbecco's Phosphate‐Buffered Saline (DPBS) and fresh cultivation medium was added for further cultivation.

### Cell Spreading Behavior

For cell spreading analysis, BJ cells expressing tdTomato‐farnesyl were embedded in either pure silk, pure collagen‐I or composite hydrogels and imaged using a DMI8 confocal microscope (Leica). 100 µm z‐stacks were acquired and collapsed to a summed slice Z‐projection image.

### Cytoskeletal Staining

For the cytoskeletal staining, C2C12 tissue constructs were fixed in 3.7% paraformaldehyde (PFA) in PBS for 30 min at room temperature, followed by washing two times with PBS and a permeabilization in 0.1% Triton X‐100 in PBS for 10 min at room temperature. After permeabilization, samples were incubated with 2 µg mL^−1^ Rhodamine‐Phalloidin in PBS for 35 min in the dark, washed twice with PBS, and stored in PBS until imaging using a Leica DMi8 confocal microscope equipped with a Leica TCS SP8 laser.

### Immunofluorescence Staining

For immunofluorescence staining, cardiac tissue constructs were first fixed with 4% formalin for 2 h at room temperature. Samples were washed with DPBS, permeabilized with 0.5% Triton X‐100 (30 min), incubated in blocking buffer (0.2% Tween‐20, 5% BSA in PBS, 60 min), followed by incubation with primary antibody against sarcomeric α‐actinin (Abcam, ab9465, 1:500) for 48 h at 4 °C. Samples were washed with 0.1% Nonidet P‐40 and incubated with secondary antibody anti‐mouse AlexaFluor 647 (Invitrogen, A‐21235, 1:500) overnight at 4 °C. Samples were stained with Hoechst33421 (10 µg mL^−1^) for 30 min at room temperature and placed on glass slides inside plastic spacers, covered with Fluoromount G and mounted with cover glass. Stained tissue constructs were imaged using a Zeiss LSM800 confocal microscope. For immunofluorescence staining, C2C12 following the cytoskeletal staining tissue constructs were fixed with 3.7 % paraformaldehyde (PFA) in PBS for 30 min at room temperature, washed twice with PBS and subsequently permeabilized in 0.1% Triton X‐100 in PBS for 10 min at room temperature. Unspecific binding sites were blocked with 5 % bovine serum albumin (BSA) and 300 mM glycine for 30 min and at room temperature. Between these steps, constructs were washed once with PBS. The primary antibody against mouse myosin (Abcam, ab51263) was diluted to 1 ng mL^−1^ in 0.1% BSA and incubated over night at 4 °C. The samples were washed and incubated with 1 ng mL^−1^ secondary goat anti‐mouse AlexaFluor 488 (Abcam, ab150113) and 1 µg mL^−1^ of 4′,6‐diamidino‐2‐phenylindole (DAPI) for 60 min in the dark at room temperature. Constructs were washed twice with PBS and stored in PBS until imaging using a Leica DMi8 confocal microscope equipped with a Leica TCS SP8 laser.

### Calcium Handling of Cardiac Tissues

Analysis of calcium handling of engineered cardiac tissues was performed using Fluo‐4 Direct Calcium Assay Kit (ThermoFisher Scientific), as described.^[^ ^24b^ ^]^ Following the manufacturer's instructions, tissue constructs were incubated for 60 min with the labeling solution diluted in culture media (1:3 dilution). Subsequently, the fluorescent movies were acquired using a live cell imaging setup (Keyence Microscopy) at 4x objective with a GFP filter at 488 nm. For each movie, three regions of interest were identified and a Z‐axis profile was generated using Fiji software. The intensity of fluorescent signal was then normalized based on the following equation:

(2) $$F=\frac{F_{i}-F_{min}}{F_{max}-F_{min}}$$

where *F_i_* is individual intensity values, *F_min_* the minimum calcium intensity and *F_max_* is the maximum fluorescent intensity in the analyzed movies.

### Statistical Analysis

Data were compiled in Microsoft Excel. Data visualization and statistical analysis were performed using Origin2020b 9.7.5 (OriginLab Corporation, Nothampton, Massachusetts USA) and GraphPad Prism 5 (Graph‐Pad Software, San Diego, California USA). Differences between multiple groups and different treatments were analyzed using a one‐way ANOVA followed by a Tukey‐test to compare means of groups with one independent variable or a two‐way ANOVA followed by post‐hoc analysis according to Dunnett. Data are presented as mean ± SD or mean ± SEM, as indicated. Reported n‐numbers denote the number of individual samples, all derived from ≥3 independent experiments.

## Conflict of Interest

T.S. is a co‐founder and shareholder of the company AMSilk GmbH. All other authors have no conflict of interest.

## Author Contributions

X.J.N. and T.U.E. contributed equally to this work. Conceptualization: F.B.E. and T.S. Methodology: F.B.E., T.S., T.U.E., and X.J.N., Validation: X.J.N., T.U.E., C.R., and M.F. Formal analysis: T.U.E., X.J.N., and C.R. Investigation: X.J.N., T.U.E., C.R., V.T.T., M.F., K.R., Z.L., and P.S. Resources: T.S., F.B.E., and G.L. Data Curation: T.S. and F.B.E. Writing‐Original Draft: F.B.E., X.J.N., and T.U.E. Writing – Review & Editing: All authors. Visualization: F.B.E., X.J.N., T.U.E., and K.R. Supervision: F.B.E., T.S., and X.J.N. Project administration: F.B.E. and T.S. Funding acquisition: T.S., F.B.E., and K.R.

## Supporting information



Supporting Information

Supplemental Movie 1

Supplemental Movie 2

Supplemental Movie 3

Supplemental Movie 4

Supplemental Movie 5

Supplemental Movie 6

Supplemental Movie 7

Supplemental Movie 8

## Data Availability

The data that supports the findings of this study are available from the corresponding authors upon reasonable request.
