# Supplementary material for: Enhancing Form Stability: Shrink‐Resistant Hydrogels Made of Interpenetrating Networks of Recombinant Spider Silk and Collagen‐I
Source: Adv Healthc Mater. 2025 Mar 27;14(12):2500311. doi: 10.1002/adhm.202500311 (PMC12057611; doi:10.1002/adhm.202500311)
Supplement: Supplementary file 1 — Supporting Information [file ADHM-14-0-s005.docx]

Supporting Information

**Enhancing Form Stability: Shrink-Resistant Hydrogels Made of Interpenetrating Networks of Recombinant Spider Silk and Collagen-I**

*Xuen J. Ng, Tilman U. Esser, Vanessa T. Trossmann, Christoph Rudisch, Maren Fiedler, Kaveh Roshanbinfar, Zan Lamberger, Philipp Stahlhut, Gregor Lang, Thomas Scheibel, Felix B. Engel*

**Supplementary Figures**


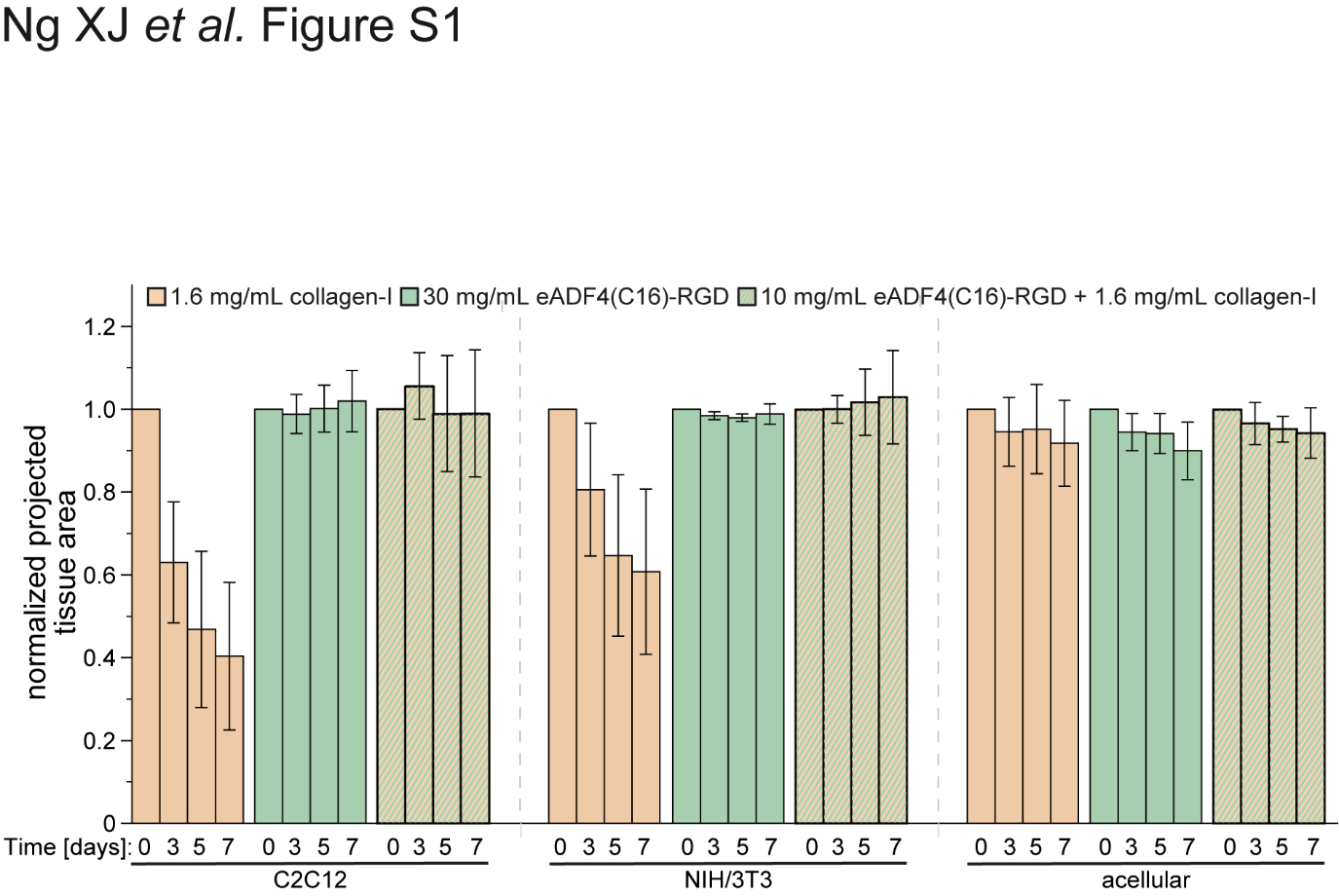


**Figure S1: Hydrogels consisting of 10 mg/mL eADF4(C16)-RGD/ 1.6 mg/mL collagen-I Spider silk/collagen-I are shrink-resistant.** Quantitative longitudinal analysis of compaction of indicated hydrogels by C2C12 myoblasts, NIH/3T3 fibroblasts in comparison to acellular hydrogels for 7 days post-fabrication. Data is mean ± SD.


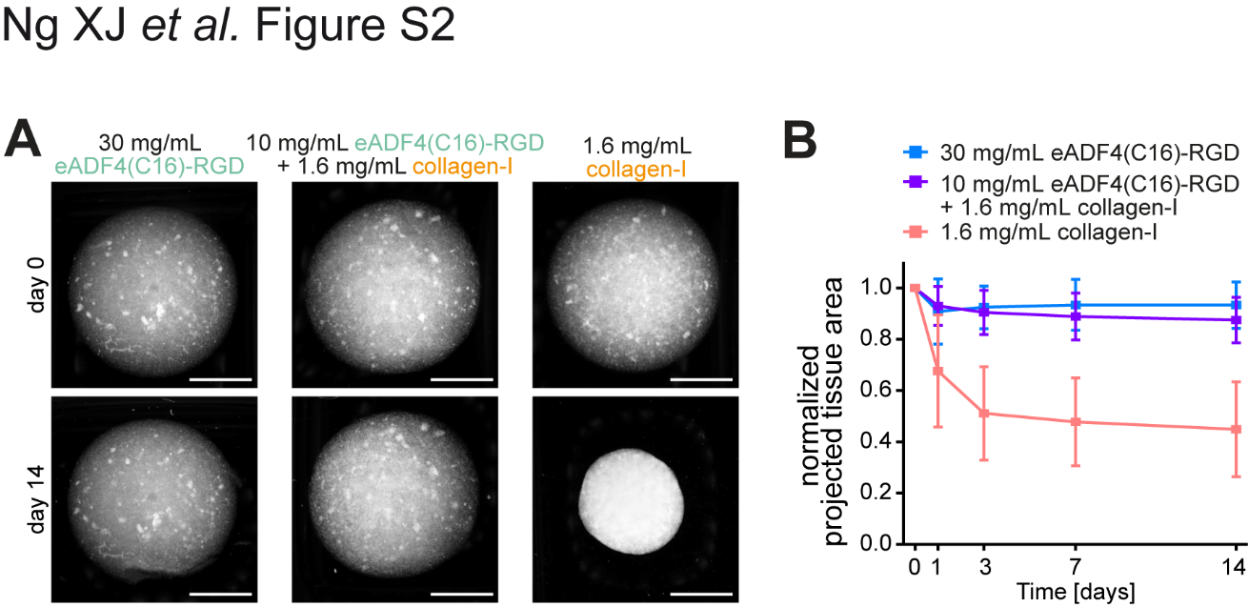


**Figure S2: eADF4(C16)-RGD/collagen-I hydrogels are suitable for cardiac tissue engineering.** (**A**) Representative darkfield images of drop-cast hydrogels containing 25 x 10^6^ hiPSC-cardiomyocytes on day 0 and 14 post-fabrication. Scale bars: 2 mm. (**B**) Quantitative analysis of compaction of tissues in A (n = 35-72). Data: mean ± SD.

**Table S1: Currently available collagen-I-based IPN hydrogels**

| **Primary** | **Secondary** | **Chemistry** | **Storage (G’)** | **Youngs (E)** | **Shrinkage** | **Properties** | **Ref.** |
| --- | --- | --- | --- | --- | --- | --- | --- |
| **network** | |  | **modulus (kPa)** | |  |  |  |
| HA (GA-func.) | collagen-I | photo-crosslinkingphysical | ~ 0.156 -0.378 | 0.468 - 1.134* | 60%, day 24 (fibroblast) | 3D printable, hiPSC-CM | ^[46]^ |
| silk fibroin | collagen-I | physical | ~ 5 - 8.1 | ~15 - 24.3* | 40%, day 14 (BMSCs) | chondro-genic differ-rentiation | ^[26]^ |
| silk fibroin | collagen-I | physical | 0.017 - 6.81 | ~ 0.051 - 20.43 | counter-acted shrinkage (hMSCs were grown on top of the hydrogels) | no analysis regarding shrinkage of cell-laden hydrogels | ^[16]^ |
| Matrigel | collagen-I | physical | - | ~ 15 - 40 | described  (valve interstitial cells) | no systematic analysis | ^[47]^ |
| alginate  (RGD-func.) | collagen-I | physical | ~ 0.03 - 1 | ~0.09 - 3* | ND  (fibroblast mor-phology/ genetic program) | effect of ma-trix stiffness on wound healing | ^[48]^ |
| CS-MA/  HA-MA | collagen-I | photo- crosslinkingphysical | - | 15 - 40 | ND | rabbit arti-cular chon-drocytes | ^[49]^ |
| Gellan gum | collagen-I | physical | ~ 1.3 - 2.4 | ~3.9 - 7.2* | ND  (ADSCs) | wound dressing | ^[50]^ |
| Trehalose and  Gellan gum | collagen-I | physical | ~ 1 | ~ 3* | ND  (ADSCs) | cryo preservation | ^[51]^ |

HA: hyaluronic acid; GA: gallic acid; func.: functionalized; CS: chondroitin sulfate; MA: methacrylate; ND: not determined; * conversion shear modulus; BMSCs: bone marrow stem cells; CM: cardiomyocytes; hMSCs: human mesenchymal stem cells; ADSCs: adipose-derived stem cells

**Table S2: Currently available silk fibroin-based IPN hydrogels**

| Primary | Secondary | Chemistry | Shear | Elastic | Total biopolymer | Shrink-age | Cell type | Ref. |
| --- | --- | --- | --- | --- | --- | --- | --- | --- |
| network | |  | modulus (kPa) | | concentration |  |  |  |
| silk fibroin | gelatin | enzymatic crosslinking | ~ 0.01 - 0.77 | ~ 0.02 - 0.23* | 15 - 40 mg/mL | ND | primary chondrocytes and htMSC | ^[52]^ |
| silk fibroin | collagen-I | physical | ~ 5 - 8 | ~15 – 24* | 8.8 - 22.3 mg/mL | ND | bone marrow stem cells | ^[26]^ |
| silk fibroin | HA | enzymatic crosslinking | n.a. | ~ 1.9 -5.1 | 40 mg/mL | ND | infrapatellar fat pad-derived MSCs | ^[53]^ |
| silk fibroin | alginate | physical | n.a. | ~ 5 - 50 | 40 - 110 mg/mL | ND | D3 mouse embryonic stem cells | ^[54]^ |
| GelMA | silk fibroin | photo-crosslinking | n.a. | ~2,5 - 70 | 80 mg/mL | ND | NIH3T3 fibroblasts | ^[55]^ |

HA: hyaluronic acid; MA: methacrylate; * conversion shear modulus; MSCs: mesenchymal stem cells; ND: not determined

**Supplementary Movie Captions**

Supplementary Movie S1: day 7.

Supplementary Movie S2: day 14.

Supplementary Movie S3: drug treatment.

Supplementary Movie S4: calcium handling.

Supplementary Movie S5: day 28.

Supplementary Movie S6: day 56 with overview on the left.

Supplementary Movie S7: day 56.

Supplementary Movie S8: day 98.
